# Distinguishing Signal From Noise in Immunopeptidome Studies of Limiting-Abundance Biological Samples: Peptides Presented by I-A^b^ in C57BL/6 Mouse Thymus

**DOI:** 10.3389/fimmu.2021.658601

**Published:** 2021-04-29

**Authors:** Padma P. Nanaware, Mollie M. Jurewicz, Cristina C. Clement, Liying Lu, Laura Santambrogio, Lawrence J. Stern

**Affiliations:** ^1^ Department of Pathology, University of Massachusetts Medical School, Worcester, MA, United States; ^2^ Department of Radiation Oncology, Weill Cornell Medicine, New York, NY, United States; ^3^ Department of Biochemistry and Molecular Pharmacology, University of Massachusetts Medical School, Worcester, MA, United States

**Keywords:** antigen presentation, MHC protein, peptide processing, mass spectrometry, thymic selection

## Abstract

Antigen presentation by MHC-II proteins in the thymus is central to selection of CD4 T cells, but analysis of the full repertoire of presented peptides responsible for positive and negative selection is complicated by the low abundance of antigen presenting cells. A key challenge in analysis of limiting abundance immunopeptidomes by mass spectrometry is distinguishing true MHC-binding peptides from co-eluting non-specifically bound peptides present in the mixture eluted from immunoaffinity-purified MHC molecules. Herein we tested several approaches to minimize the impact of non-specific background peptides, including analyzing eluates from isotype-control antibody-conjugated beads, considering only peptides present in nested sets, and using predicted binding motif analysis to identify core epitopes. We evaluated these methods using well-understood human cell line samples, and then applied them to analysis of the I-A^b^ presented immunopeptidome of the thymus of C57BL/6 mice, comparing this to the more easily characterized splenic B cell and dendritic cell populations. We identified a total of 3473 unique peptides eluted from the various tissues, using a data dependent acquisition strategy with a false-discovery rate of <1%. The immunopeptidomes presented in thymus as compared to splenic B cells and DCs identified shared and tissue-specific epitopes. A broader length distribution was observed for peptides presented in the thymus as compared to splenic B cells or DCs. Detailed analysis of 61 differentially presented peptides indicated a wider distribution of I-A^b^ binding affinities in thymus as compared to splenic B cells. These results suggest different constraints on antigen processing and presentation pathways in central versus peripheral tissues.

## Introduction

MHC-II antigen presentation pathways have been explored mostly in professional antigen-presenting cells (APC) such as B cells and dendritic cells, because of their important role in generating and regulating immune responses. That work has been aided by robust MHC-II expression in these cells. However, MHC-II is expressed by other cells types including epithelial cells in several tissues. Of these, thymic epithelial cells are of particular interest because of their role in T cell selection (1, 2). AIRE expression by medullary thymic medullary epithelial cells provides a regulated mechanism for stochastic expression of genes usually expressed in a tissue-specific fashion and is required for efficient T cell negative selection (3, 4). In addition to thymic epithelial cells, migratory DC and B cells also are known to bring tissue-specific proteins and processed peptides into the thymus for negative selection (5–7). Pioneering differential peptidome analysis by Marrack et al., showed that the major peptides presented by MHC-II in the thymus also were associated with MHC-II in the spleen (8). This was somewhat surprising given the idea that efficient T cell negative selection against all possible self-antigens depends on antigen presentation in the thymus of peptides derived from tissue-specific gene products, many of which would not be expected to be expressed in the spleen. However, due to experimental sensitivity constraints at that time, few peptides overall (<20) were identified, and many low-abundance peptides likely evaded detection. Subsequent work on peptides presented by MHC-II in human thymus reported substantially more peptides (~120) (9, 10), with experimental validation of thymic presentation of several AIRE-dependent transcripts (10). Self-peptides broadly expressed in most tissues also were well-represented in the thymic MHC-II peptidomes, as expected from earlier work on murine MHC-I and CD8 T cells showing that expression of such peptides was required for positive selection of developing thymocytes (11, 12). Challenges in analyzing thymic immunopeptidomes include presence of large numbers of non-MHC expressing cells in same tissue, and relatively low expression levels on cells that do express MHC-II. Much of the technology development for immunopeptidome analysis has grown out of analysis of cell lines and highly enriched samples, and strategies for dealing with analysis of limiting abundance biological samples are being developed (13–17).

Here, we evaluated ways to address these issues in a comparative analysis of the MHC-II peptidomes expressed in thymus and spleen of C57BL/6 mice. This strain of mice expresses only a single MHC-II molecule, I-A^b^, simplifying assignment of eluted peptides to MHC molecules, and is a mainstay of antigen presentation and T cell selection studies. Previous studies of these mice reported analysis and quantitation of ~150 peptides eluted from splenic B cells and dendritic cells (DC) (18), and more recently over 2700 peptides from unfractionated splenocytes (15), but to our knowledge no study utilizing modern immunopeptidome techniques to characterize the C57BL/6 thymic peptidome has been reported. The thymus consists of over 95% thymocytes, which do not express MHC-II, together with the much smaller MHC-II expressing populations that include cortical and medullary thymic epithelial cells (cTECs and mTECs) (19), resident and migratory DC, B cells, macrophages, and a variety of endothelial cells, neural crest-derived pericytes and mesenchymal cells (20–24). Although one of the human thymus studies fractionated this population into DC and non-DC populations using CD11c-bead enrichment (9), because of the small size of mouse thymi and substantial losses that we encountered during fractionation in preliminary studies, we analyzed the various cell types in the thymus all together. We did fractionate the spleen into B cells and DC, as in a previous mouse spleen study (18). We included both natural abundance and FLT3L induced splenic DC populations; provision of FLT3L *in vivo* is common way to expand murine DC populations, analogous to human G-CSF induction therapy (25, 26). Finally, we included both resting splenic DC as well as splenic DC matured by systemic LPS administration, for comparison with antigens presented by DC in the thymus where DC in various activation states are believed to contribute to thymic selection (3, 27).

We explored several methods to mitigate the effects of the low abundance of MHC-II-expressing cells in the thymus, including characterization of peptides captured by non-specific immunoaffinity and control resins, enrichment of membrane and vesicular compartments prior to isolation, and consideration of MHC-II length preferences and antigen presentation pathways leading to nested sets of peptides sharing a core epitope. These methods proved crucial to obtaining reliable information on thymic peptidomes. We found that the thymic I-A^b^-presented peptidome was characterized by a broader distribution of lengths and binding affinities than the splenic B cell peptidome or splenic DC peptidomes, suggestive of possible differences in antigen processing pathways.

## Materials and Methods

### Isolation of Splenic B Cells From C57BL/6 Mice

Spleens were isolated from C57BL/6 mice and were dissociated into single-cell suspensions by treatment with collagenase type II enzyme (Sigma-C6885), in order to facilitate separation from extracellular matrix and other unwanted tissue-derived material that might mask the ionization of low abundant peptides, and to promote detergent solubilization. Splenic B cells were evaluated for I-A^b^ expression by gating on the B220^+^ CD43^-^ CD11b^-^ population. The cells were blocked with 50 µg/ml anti-mouse CD16/CD32 (2.4G2, BioXCell, West Lebanon, NH) prior to staining. Cells were acquired on an LSR II flow cytometer (Becton Dickinson) and analyzed using FlowJo version 9.8.5 software (Tree Star, Ashland, OR). To isolate mature B cells from the splenocyte population, CD43^-^ and CD11b^-^ expressing cells were depleted using biotinylated anti-mouse CD43 and CD11b (BioLegend) in conjunction with the EasySep Mouse Streptavidin RapidSpheres Isolation Kit (Stem Cell Technologies, Cambridge, MA) according to the manufacturer’s instructions. Purity post-isolation was determined by FACS to be >90% for each sample, with an average purity of 94 ± 2.6%. Splenic B cells were isolated from 9 mice for each replicate sample.

### Isolation of Dendritic Cells From C57BL/6 Mice

Splenic DC were isolated from C57BL/6 mice either untreated or after *in vivo* expansion of DC using Flt3L (**Table 1**). For Flt3L treatment, C57BL/6 mice were injected subcutaneously with 40 x 10^6^ B16-FLt3L-producing melanoma cells and humanely sacrificed 12-14 days later. (Clement et al., 2016a). For LPS treatment, mice were injected intraperitoneally with 0.5mg/kg and humanely sacrificed 6 hours later. Spleens were isolated and dissociated into single-cell suspensions, and DC populations were enriched by negative selection using magnetic beads, or by 30% bovine albumin solution density-gradient centrifugation (Sigma-Aldrich). As determined by flow cytometry, after enrichment the preparation routinely contains 70 to 80% CD11c^+^ DCs with intermediate MHC-II levels, and includes both the CD8α^+^ and CD8α^-^ populations (13). Splenic DCs were isolated from 1-6 mice for each replicate sample of Flt3L-treated mice, or from 20 untreated mice.

**Table 1:** I-A^b^ preparations Summary of the I-A^b^ peptides eluted from four biological replicates of thymus, three biological replicates of splenic B cells, four different preparations of splenic DCs, and four different amounts of human LG2 cells.

Preparation	Number of mice used for tissue preparation	Number of cells used for I-A^b^ isolation	Amount of I-A^b^ isolated (µg)	Total number of peptide sequences identified	Number of peptides: ≥3 peptides/core
Splenic B #1	9	200 million	22	909	404
Splenic B #2	9	100 million	10.5	507	316
Splenic B #3	9	100 million	8.5	510	300
Thymus #1	3	200 million	14	622	256
Thymus #2	3	100 million	3.5	391	125
Thymus #3	3	100 million	7	481	187
Thymus #4	3	100 million	4	418	160
DC #1 (Flt3L)	1	140 million	11	570	280
DC #2 (Flt3L+LPS)	1	70 million	11.2	532	295
DC #3 (untreated)	20	247 million	8	833	425
DC #4 (Flt3L)	6	244 million	17.5	1180	618
LG2 cells	–	10 million	9.4	1422	752
LG2 cells	–	1 million	1.1	643	296
LG2 cells	–	100 thousand	0.107	179	37
LG2 cells	–	50 thousand	0.062	85	3

### Isolation of Thymus Cells

Thymi were isolated from C57BL/6 mice were dissociated into single-cell suspensions using collagenase type II enzyme (Sigma-C6885). Total thymic cell populations from 3 mice were used for each of the four replicate samples. For three of these samples, splenic B cells were prepared from the same sets of mice, and processed in parallel with the thymic samples, although to increase cell numbers the B cell samples also included spleens from additional mice (see **Table 1**).

### Culture and Isolation of Human B Cells

The human EBV (Epstein Barr virus) transformed B lymphoblastoid cell line LG-2 (28), which is homozygous DRA1*01:01, DRB1*01:01, was cultured in RPMI medium with 10% fetal calf serum. Cells were pelleted at 400 × g and washed in PBS for 2 times before analysis.

### ELISA Assays for I-A^b^ and HLA-DR1

MHC levels samples of the initial cell lysates prior to immunoaffinity purification were measured by ELISA assay to estimate the total MHC present, and the flow-through portions from the various immunoaffinity steps were measured to evaluate stepwise recoveries (**Supplementary Table S1**). For determination of I-A^b^, the monoclonal antibody 17/227 of 200ng/well, diluted in bicarbonate/carbonate buffer pH 9.0 was used to coat the wells of high binding 96 well plates (Immulon 4 HBX-ND541225). The plates were incubated at 4°C for O/N or at 37°C for 2hrs. The wells were blocked using 3% BSA in 1X PBS for 1hr at 37°C. The recombinantly purified I-A^b^-peptide complex was used as standards protein to calculate the amounts of I-A^b^ in each preparation. The standards ranging from 2ug-1ng were used. The monoclonal anti-murine MHCII antibody (M5/114) was used as the primary antibody for the detection. HRP-conjugated goat anti-rat IgG (KPL:14-16-12) was used as the secondary antibody followed by the ABTS substrate solution (Roche-11 684 302 001) for the colorimetric detection. Incubations were done at 1hr at 37°C and the washes between every incubation was performed using 1X PBST buffer (1X PBS, 0.05% triton X-100) three times. The dilutions of protein and antibody was done in dilution buffer 0.3% BSA, 0.1% Triton-X100 in 1X PBS. The HLA-DR1 ELISA assay was performed essentially as described (29). The procedure is similar to that for I-A^b^, except that mouse anti-HLA-DR monoclonal antibody LB3.1 (30), polyclonal anti-HLA-DR1, and HRP-conjugated goat anti-rabbit IgG (Life technologies) were used as capture, primary, and secondary antibodies, respectively.

### Preparation of Affinity Resins

M5/114 antibody was coupled to CNBr-activated sepharose 4B beads. M5/114 antibody was purified using hybridoma and dialyzed using 0.1M NaHCO_3._ The concentration of the antibody used for the coupling was ~1-10mg/ml. We used 5mg of antibody for coupling to 1 ml of the CNBr-activated sepharose 4B beads. The beads were washed rapidly with coupling buffer- 0.1M NaHCO_3_, 0.5M NaCl pH 8.5. The beads were then mixed with the antibody slowly on the rotor at RT for 1 h. The beads were then thoroughly washed with the coupling buffer and blocked the remaining active groups on beads by incubating the antibody conjugated beads with blocking buffer (0.1M glycine in coupling buffer, pH 8.5) for 2 h at RT. The beads are then washed with alternating cycles of coupling buffer and the acetate buffer (0.1M sodium acetate, 0.5M NaCl, pH 4.0). A isotype control antibody (IgG2b) resin was prepared similarly. LB3.1 and isotype control antibody was coupled to recombinant Staphyloccal Protein A agarose-based resin (IPA300S, Repligen, Cambridge MA) using dimethyl pimelimidate coupling essentially as described (28). The antibody-conjugated beads were stored in 1X PBS buffer containing 0.02% sodium azide until the further usage.

### Isolation of MHC-Peptide Complexes

For isolation of I-A^b^ and HLA-DR1 peptide complexes we used a sequential immunoaffinity chromatography protocol previously developed for biochemical studies, optimized for sample yield and purity, and used in many immunopeptidome characterizations (28). The procedure employs pre-columns with unconjugated beads as well as beads conjugated with isotype control antibody. These are intended to remove peptides, proteins, and other contaminants that can bind to the agarose substrate, protein A capture ligand if present, or immobilized antibodies outside of their combining site, for example Fc receptors abundant on B cells and DC. In preliminary experiments we eluted peptides from these columns separately and found distinct sets of non-specifically-bound peptides, so we included both types of pre-columns, although in most experiments peptides eluted from these columns were pooled. Four independent samples of thymus, three independent samples of splenic B cells and dendritic cells from four different preparations were analyzed for I-A^b^ peptidomes. In other experiments samples containing various numbers of LG2 cells were analyzed for HLA-DR1 peptidomes. Cell pellets were resuspended in 50 mM Tris-HCl, 150 mM NaCl, pH 8.0, containing protease inhibitor cocktail (Sigma-P2714) and 5% β-octylglucoside, freeze-thawed for 5-6 times. The lysate was spun at 4000×g for 5 min at 4°C to remove the cellular debris. The supernatant was collected and further spun using ultracentrifuge at 100,000 × g for 1 h at 4°C. In some experiments, total cell membranes were solubilized instead of whole cell pellet. Cells were suspended in ice-cold hypotonic buffer (10 mM Tris-HCl, pH 8.0, containing protease inhibitors). Repeated (4–5) freeze-thaw cycles were used for cell disruptions and in between the cycles the cells were homogenized using dounce homogenizer with 10 strokes each cycle. Cellular debris was removed by centrifuging the lysate at 4000 × g for 5 min at 4°C. The supernatant was collected and further centrifuged at 100,000 × g for 1 h at 4°C to pellet the membrane fraction. The membrane pellet was solubilized in ice-cold 50 mM Tris-HCl, 150 mM NaCl, pH 8.0, containing protease inhibitors and 5% β-octylglucoside, and then mixed slowly overnight on shaker at 4°C. Supernatant containing the solubilized membrane was recovered by centrifuging the lysate at 100,000 × g for 1 h at 4°C. The supernatant was used for the isolation of the MHCII-peptide complexes using an immunoaffinity column of M5/114 monoclonal antibody immobilized onto CNBr activated Sepharose CL-4B using following steps. The lysates were then allowed to mix with the CNBr activated Sepharose beads only, followed by isotype control antibody conjugated beads slowly on shaker for 1 h at 4°C to prevent nonspecific binding of proteins to beads. This equilibrated lysate was incubated with M5/114 conjugated beads and allowed to mix slowly for 2 h on shaker at 4°C. The column was washed by passing several buffers in succession as follows: (1) 50 mM Tris-HCl, 150 mM NaCl, pH 8.0, containing protease inhibitors and 5% β-octylglucoside (5 times the bead volume); (2) 50 mM Tris-HCl, 150 mM NaCl, pH 8.0, containing protease inhibitors and 1% β-octylglucoside (10 times the bead volume); (3) 50 mM Tris-HCl, 150 mM NaCl, pH 8.0, containing protease inhibitors (30 times the bead volume); (4) 50 mM Tris-HCl, 300 mM NaCl, pH 8.0, containing protease inhibitors (10 times the bead volume); (5) 1X PBS (30 times the bead volume); and (6) HPLC water (100 times the bead volume). Recovery of MHC-peptide complexes at various steps in the procedure was assessed by ELISA assay as described above (**Supplemental Table S1**). MHC proteins were released from the resin and HLA-DR1/I-A^b^ peptides eluted using 2% TFA (The Nest Group, USA). Eluted peptide mixtures were then separated from MHC proteins, residual detergent, and cellular material by binding to a Vydac C4 macrospin column and eluting with 30% acetonitrile containing 0.1% (v/v) TFA. Solvent was removed by Speed-Vac and the dried peptide extracts were stored at -80° C or used immediately.

### Analysis of Peptides Eluted From I-A^b^ or HLA-DR1

Peptide extracts were reconstituted in 25 µl 5% acetonitrile containing 0.1% (v/v) trifluoroacetic acid and separated on a nano-ACQUITY (Waters Corporation, Milford, MA) UPLC with technical triplicate injections. In brief, a 3.0 µl injection was loaded in 5% acetonitrile containing 0.1% formic acid at 4.0 µl/min for 4.0 min onto a 100 µm I.D. fused-silica precolumn packed with 2 cm of 5 µm (200Å) Magic C18AQ (Bruker-Michrom, Auburn, CA) and eluted using a gradient at 300 nL/min onto a 75 µm I.D. analytical column packed with 25 cm of 3 µm (100Å) Magic C18AQ particles to a gravity-pulled tip. The solvents were A) water (0.1% formic acid); and B) acetonitrile (0.1% formic acid). A linear gradient was developed from 5% solvent A to 35% solvent B in 90 min. Ions were introduced by positive electrospray ionization *via* liquid junction into a Q Exactive hybrid mass spectrometer (Thermo Fisher Scientific). Mass spectra were acquired over m/z 300–1750 at 70,000 resolution (m/z-200), and data-dependent acquisition (DDA) selected the top 10 most abundant precursor ions in each scan for tandem mass spectrometry by HCD fragmentation using an isolation width of 1.6 Da, collision energy of 27, and a resolution of 17,500.

### Peptide Identification

Raw data files were peak processed with Proteome Discoverer (version 2.1, Thermo Fisher Scientific) prior to database searching with Mascot Server (version 2.5, Matrix Science, Boston, MA) against the combined database of UniProt_Mouse which was downloaded on 10/7/16 with 57,984 entries. Search parameters included “no enzyme” specificity to detect peptides generated by cleavage after any residue. The variable modifications of oxidized methionine and pyroglutamic acid for N-terminal glutamine were considered. The mass tolerances were 10 ppm for the precursor and 0.05Da for the fragments. Search results were then loaded into the Scaffold Viewer (Proteome Software, Inc., Portland, OR) for peptide/protein validation and label-free quantitation. Scaffold assigns probabilities using PeptideProphet or the LDFR algorithm for peptide identification and the ProteinProphet algorithm for protein identification, allowing the peptide and protein identification to be scored on the level of probability. An FDR of 1% was adjusted for reliable identification of peptides.

### Label-Free Quantitation

Label-free relative quantitation of all peptides eluted from thymic cells and splenic B cells was performed using precursor intensity analysis in Scaffold, Scaffold Q&/Q&S. Scaffold uses the precursor intensity information from the Thermo Proteome Discoverer. The software normalizes total precursor intensity values across the samples and calculates fold change or log2 normalized intensity across the samples while considering different statistical parameters like t test, ANOVA and coefficient of variance. The log2 normalized intensity values were converted to intensities for subsequent analyses. Triplicate technical replicates were run for each sample and the single average value was used to represent the three technical replicates. For analysis of core epitope intensities, the intensity values for all peptides sharing the same core epitope were summed within each technical replicate. The core epitopes were predicted using the NetMHCIIpan algorithm. The technical replicates were averaged for core epitopes as described above for peptides.

### Gibbs Clustering

GibbsCluster-2.0 (31) was used to align the eluted peptide sequences and analyze the motifs, which were displayed with Seq2Logo 2.0 (32). We allowed the software to include cluster sizes of 1-5 with a motif length of 9 amino acids and clustering sequence weighting. Default values were used for other parameters: number of seeds =1, penalty factor for inter-cluster similarity =0.8, small cluster weight = 5, no outlier removal, iterations per temperature step =10, Monte Carlo temperature =1.5, intervals for indel, single peptide and phase-shift moves = 10, 20, and 100, respectively, and Uniprot amino acid frequencies were used. For each sample, we selected the cluster that included the largest number of peptides analyzed. In most cases a single cluster included most of the sequences, but for the total thymus sample (**Figure 2D**) two clusters were required. A preference for hydrophobic residue at P1 was used to align the motifs at the P1 position. The fraction of sequences that contributed to each cluster is shown in the figures.

### Expression and Purification of I-A^b^-3R Complex for Binding Affinity Measurements

The soluble I-A^b^-3R, a mouse MHC-II protein contains α and β subunit. The peptide 3R (FEAFMARAKAAV) was engineered at its C-terminus on the beta subunit and expressed from a baculovirus plasmid p3288 I-A^b^-BirA. The I-A^b^-3R protein complex is about 62kDa molecular weight. The I-A^b^-3R was expressed in Hi5 cells in a shake flask to a density of 2 million cells/ml in a mixture of 70% EX-CELL 405 serum free medium for insect cells (Sigma, cat. #14405C) and 30% complete graces medium (Thermo Fisher Scientific, cat. #11605-094) in presence of antimycotic. The cells were further infected using I-A^b^-3R virus and incubated for 5 days in a 27°C at 100 rpm. Post-incubation, the supernatant was collected and filtered through a 0.45µm filter. Protease inhibitors (0.02% NaN3, 0.7µg/ml pepstatin, 1µg/ml leupeptin and 0.25mM PMSF) were added to supernatant and purified using M5/114 antibody conjugated Sepharose CL-4B column. The eluted purified protein was further purified using Superdex 200 gel filtration column.

### Peptide Synthesis and Labeling

The CLIP peptide Ac-VSKMRMATPLLMQ were synthesized (21st Century Biochemicals, Marlboro, MA) and labeled with Alexa Fluor 488 tetrafluorophenyl ester (Invitrogen, Eugene, OR) through primary amine of lysine. For this purpose, the peptide (2 mg) was dissolved in 400 μl of sodium bicarbonate (150 mM pH 9.8) and mixed with Alexa488-tetrafluorophenyl ester (1mg) (Molecular Probes). After one-hour incubation at room temperature, labeled peptide was purified by reverse HPLC (Agilent) using a C18 column (Jupiter 300A 00G-4053-E0) and a gradient of acetonitrile in 0.02% trifluoracetic acid.

### I-A^b^-CLIP Peptide Exchange Assay

A fluorescence polarization (FP) assay was used to measure the IC50 of using N-terminally-acetylated CLIP peptide labeled with Alexa Fluor 488 tetrafluorophenyl ester (Invitrogen, Carlsbad, CA) *via* the primary amine at K3 as probe peptide as previously described (34). The binding reactions were carried in buffer conditions of 100 mM sodium citrate, 50 mM sodium chloride, 0.1% octyl β-D-glucopyranoside, 5 mM ethylenediaminetetraacetic acid, 0.1% sodium azide, 0.2 mM iodoacetic acid, 1 mM dithiothreitol). The I-A^b^-3R complex has a thrombin linker to cleave off the 3R peptide from I-A^b^ protein. Thrombin is added during all the reactions at a concentration of 1U/ug and inactivated after 3hrs of reaction using 0.1mM phenylmethanesulfonyl fluoride. The I-A^b^-3R concentration used was selected by titrating I-A^b^-3R against fixed labeled peptide concentration (25 nM) and choosing the concentration of I-A^b^-3R that showed ~50% maximum binding. For calculating IC50 values, 100 nM I-A^b^-3R was incubated with 25 nM Alexa488-labeled CLIP probe peptide, in combination with a serial dilution of test peptides, beginning at 100 µM followed by 5-fold dilutions in presence of 0.5µM HLA-DM. The reaction mixture was incubated at 37°C. The capacity of each test peptide to compete for binding of probe peptide was measured by FP after 72 h at 37°C. The assay was read using a Victor X5 Multilabel plate reader (PerkinElmer, Shelton, CT). FP values were converted to fraction bound by calculating [(FP_sample - FP_free)/(FP_no_comp - FP_free)], where FP_sample represents the FP value in the presence of test peptide; FP_free represents the value for free Alexa488-conjugated CLIP peptide; and FP_no_comp represents values in the absence of competitor peptide. We plotted fraction bound versus concentration of test peptide and fit the curve to the equation y = 1/(1 +[pep]/IC50), where [pep] is the concentration of test peptide, y is the fraction of probe peptide bound at that concentration of test peptide, and IC_50_ is the 50% inhibitory concentration of the test peptide.

### Source Protein Tissue Location/Gene Ontogeny Analysis

Source proteins for peptides eluted from thymus, splenic B cells and splenic DCs were analyzed for tissue sources. The proteins having 2 or more peptides were analyzed using Uniprot Consortium (35). The fraction of the total is plotted for each tissue type.

### Statistics

Specific t-tests were used for different analyses, which are indicated in the figure legends of each plot. Prism (version 7.03, GraphPad, San Diego, CA) was used for statistical analysis and graphing data.

## Results

### Isolation of I-A^b^ From Total Thymus, Splenic B Cells and Splenic Dendritic Cells

For immunopeptidome analysis, spleen and thymus were isolated from C57BL/6 mice and dissociated into single cell suspensions before detergent lysis, I-A^b^ isolation, peptide elution, and mass spectrometry. Spleen samples were fractionated into splenic B and splenic DC preparations, but whole thymus preparations were used as noted above. Triplicate preparations of splenic B, quadruplet preparations of thymus, and a total of four different preparations of splenic DC were analyzed. Splenic DC preparations included samples from normal mice, mice harboring a Flt3L secreting tumor to promote production of DC, and Flt3L treated mice injected with lipopolysaccharide to induce DC maturation. Thymii were pooled from 3 mice for each preparation, and spleens were pooled from 9 mice for each B cell preparation or from 1-20 mice for each DC preparation depending on treatment. Typical preparations contained ~10 micrograms of I-A^b^ (range 3.5-22) (**Table 1**).

### Characterization of Eluted Peptides by Mass Spectrometry

**Figure 1:**
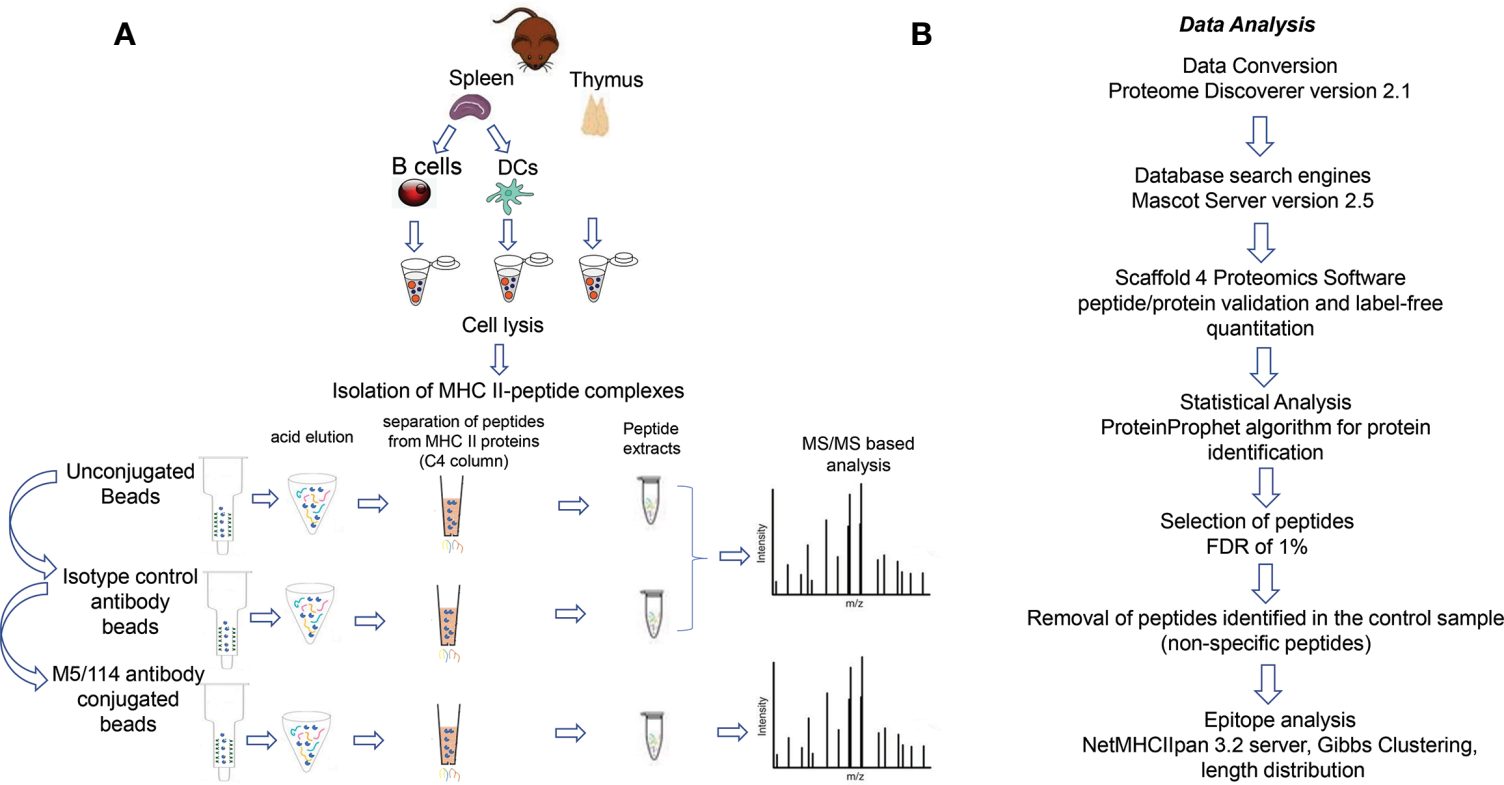
Workflow **(A)** Schematic representation of I-A^b^ peptide elution workflow from thymus, splenic B cells and splenic DCs. **(B)** Strategy for peptide identification and I-A^b^-peptidome characterization.

We used a standardized protocol to isolate the I-A^b^-presented peptides from the different tissues and cell types (see Materials and Methods and **Figure 1A**). Whole-cell detergent lysates of single-cell suspensions were prepared, non-solubilized material was removed by ultracentrifugation, and the clarified supernatants were used for isolation of I-A^b^ followed by peptide elution. We used a three-step sequential immunoaffinity procedure (28), in which the samples were incubated first with control Sepharose-CL-4B beads, then with CNBR-activated Sepharose-4B beads coupled with an isotype control antibody, and finally with CNBR-activated Sepharose-4B beads coupled with the I-A^b^-specific antibody M5/114 (**Figure 1A**). After a series of stringent washes, I-A^b^-peptide complexes were eluted using trifluoroacetic acid solution to denature the MHC-II protein. Released peptides were separated from I-A^b^ and desalted using a reverse-phase C4 column. Eluted peptides were analyzed using a standard LC/MS/MS data-dependent acquisition and analysis pipeline (see Materials and Methods and **Figure 1B**). Three biological replicates of each sample were analyzed. For each biological replicate the eluted peptide sample was split into thirds, which were injected separately as technical replicates. The peptide ion fragmentation spectra were associated with peptide sequences using Mascot Server and the UniProt_Mouse sequence database and were filtered to a 1% false-discovery rate based on the Protein Prophet algorithm. Peptide sequences identified in bead-only and the isotype control eluates were removed from the I-A^b^-specific eluates. These control-elution peptides included both high and low abundance species, and were not always observed reproducibly in biological replicates, and so peptide sequences appearing in any of the control elutions for any of the samples were removed from each of the specific elution datasets. The filtered sets of peptide sequences were further analyzed to identify predicted MHC-II core epitopes, binding motifs, and length distributions.

### Peptides Eluted From Immunoaffinity-Purified I-A^b^ Preparations From Thymus, Splenic B Cells, and Splenic Dendritic Cells of C57BL/6 Mouse

**Figure 2:**
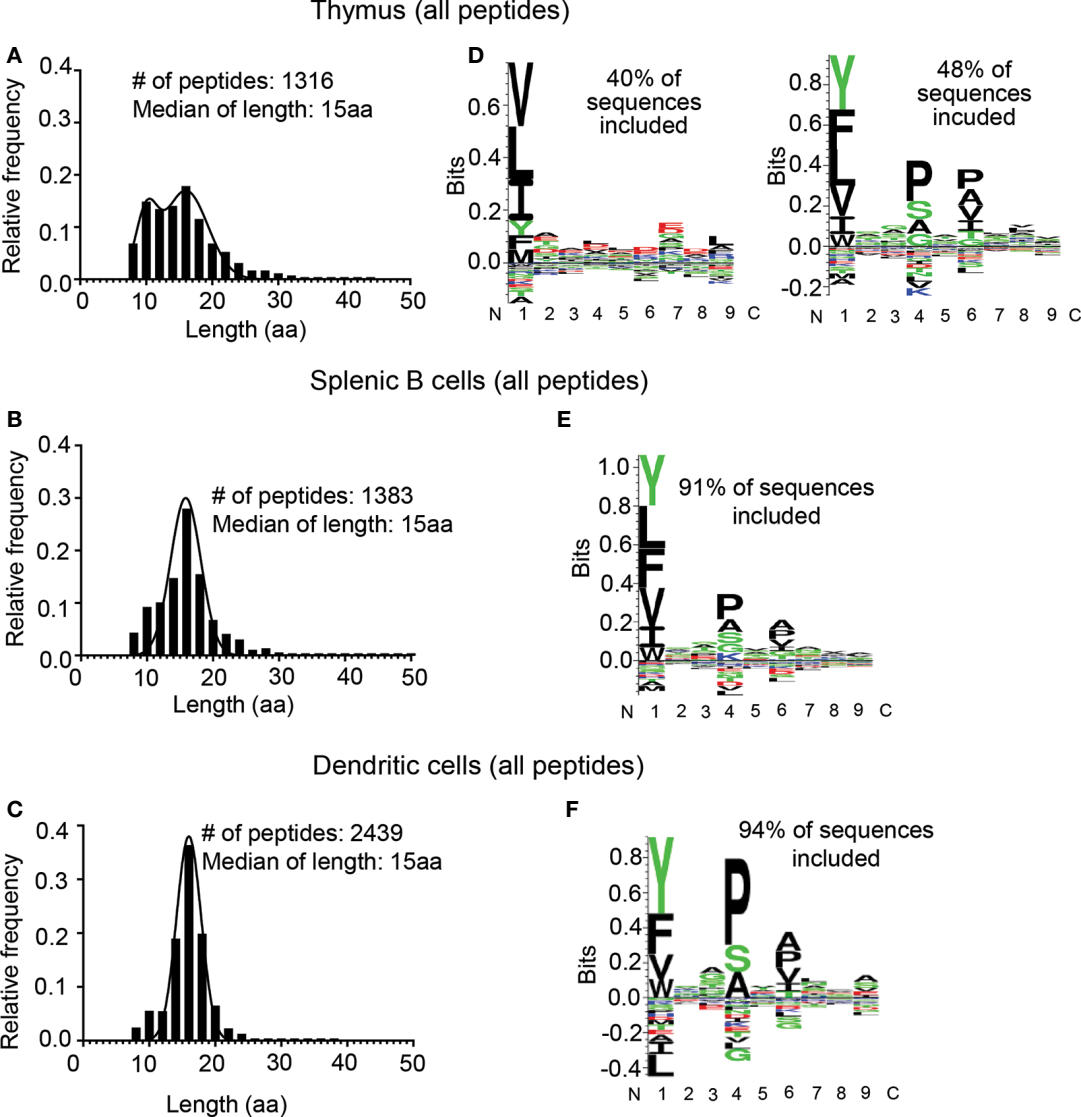
Length distribution and motif analysis for all eluted peptides from thymus, splenic B cells, and splenic DCs. **(A–C)** Length distribution; **(D–F)** motif analysis. **(A, D)** Eluted peptides from thymus; **(B, E)** from splenic B cells; and **(C, F)** from splenic dendritic cells (DCs). Motifs determined using the GibbsCluster 2.0 algorithm (33) and displayed using Seq2Logo (32); note different vertical scales. The fraction of total sequences represented by the GibbsCluster motif is shown. For the thymus sample two clusters were required to include the majority of the peptides.

Overall, we identified 1316 peptide sequences in the thymus eluates, 1383 peptides in the splenic B cell eluates, and 2439 peptides in the splenic DC eluates (**Figures 2A–C** and **Supplementary Table S2**). The thymus and splenic B cell peptides represented four and three biological replicate samples respectively. The splenic DC peptides represented four samples including conventional and Flt3-ligand induced populations from resting mice and those treated with LPS to induce DC maturation, likely accounting for the greater variety of DC sequences identified. The median peptide lengths for the three samples were similar, 15 amino acid residues (aa) for the thymic peptides, splenic B and splenic DC peptides. However, the distribution of peptides lengths was different, with the thymic peptide distribution showing two peaks, one centered at ~16 aa and another at ~10aa (**Figure 2A**), whereas the B cell and DC samples showed monomodal distributions centered around the median length of ~15aa (**Figures 2B, C**), as observed previously for naturally processed MHC-II-bound peptides from several sources (10, 15, 17). The consensus sequence motifs as identified by an unsupervised Gibbs sampling alignment/clustering algorithm (33) also differed for the thymic peptides as compared to the other sets. The splenic B and DC samples showed the expected pattern of preferences at the P1, P4, and P6 positions with a large hydrophobic residue at P1 and small residues at P4 and P6 (**Figures 2E, F**), as previously observed for other high-density I-A^b^ peptidomes from spleen, lymph nodes, and pancreas (15, 17). By contrast the thymic sample showed two clusters, one with an unexpected preference for leucine at the P1 and to a lesser extend at the P9 positions, and another that corresponded to the known I-A^b^ motif as observed in the other samples (**Figure 2D**). Although the unusual length distribution observed for the thymic peptides was suggestive of possible differences in thymus and spleen antigen processing, the cluster of sequences representing ~40% of the sample that lacked a clear I-A^b^ binding motif suggested that some of the peptides identified in this sample might not represent actual I-A^b^ binders.

### Non-Specific Peptides

To help identify characteristics of non-I-A^b^-binding peptides that might be present in the immunoaffinity-purified preparations, we analyzed the length distribution and sequences characteristics for the non-specific peptides eluted from the beads-only and isotype-antibody columns of the thymus sample preparation. The length distribution for these non-specific peptides were centered around a median of ~10 aa (**Supplementary Figure S1A** and **Supplementary Table S6**). A similar length distribution and motif analysis profile was observed for the peptides eluted from the beads only column from a variety of different mouse cell types investigated in our laboratory (**Supplementary Figure S1B**). We suspected that the peptides eluted from thymus I-A^b^ preparations might include peptides similar to the non-specific peptides eluted from the control columns, and that this might help to explain the bimodal length distribution observed for the thymus samples. Indeed, computational fractionation of the total thymus eluted peptides into pools for each length showed that the shorter peptides (8-12 aa) had non-I-A^b^-like preferences (**Supplementary Figure S2**) as also observed for one of the clusters in the full thymus sample (**Figure 2D**). Longer peptides (14-18aa) had the expected the expected I-A^b^ motif (**Supplementary Figure S2**). However, we did not want to apply an arbitrary length cutoff to the eluted peptide datasets, because we observed that the I-A^b^-specific peptide distributions from spleen included peptides shorter than 12 aa (**Figures 2B, C**), as previously reported for other published I-A^b^ immunopeptidomes, because the non-specific peptide length distributions had tails extending well beyond 12 aa (**Supplementary Figure S1A, B**), and because MHC-II antigen processing pathways in the thymus are poorly characterized and we wanted to investigate the possibility of an altered length distribution in that tissue.

In order to try to eliminate the contribution of apparently non-specific peptides in the thymus samples, we evaluated two strategies used in previous immunopeptidome characterizations. First, we tested whether isolating I-A^b^ from total cellular membrane preparations instead of whole cell lysates would provide enriched samples with lower levels of background peptides. However, the length distribution of all the peptides eluted from the membrane fraction of the thymus showed the same two peaks, one at ~10 aa and another at ~16aa, as for the thymus whole cell preparation (**Supplementary Figure S3A**). Also, the distribution of peptides eluted from the beads-only column of the membrane preparation looked similar to the beads-only eluted peptides of the whole cell preparation (**Supplementary Figure S3B**). These results indicated that a membrane fractionation strategy as used previously (13, 36, 37) was not helpful in removing the non-specific peptides. Second, we tested whether restricting the analysis to peptides identified reproducibly in every sample would help to remove non-specific peptides, with the idea that the non-specific peptides might be present sporadically as a result of low-frequency random binding or sampling events. However, considering only peptides present in all four biological replicate samples, and at least two technical replicates in each sample, did not provide cleaner thymic peptidome datasets (**Supplementary Figure S4**). Although both strategies removed many peptides from the datasets, the considerably trimmed datasets still retained many apparently non-specific peptides, and we sought other ways to identify bona-fide MHC-II binders in eluates from low-abundance samples.

### Limiting Sample Amounts Results in Increasing Proportion of Non-Specific Peptides and Masks the Presence of MHC Binders in the Sample

**Figure 3:**
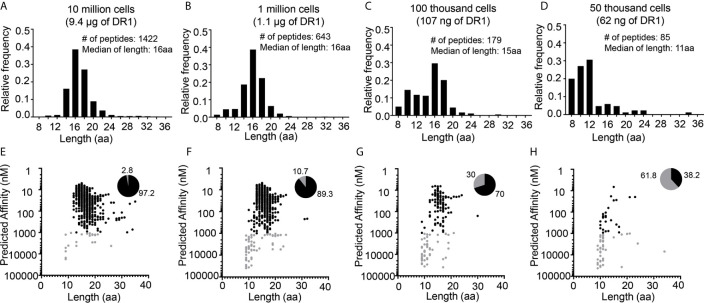
Distribution of length and predicted binding affinities for peptides eluted from different numbers of human LG2 cells. Length distribution of **(A)** 10 million, **(B)** 1 million, **(C)** 100 thousand and **(D)** 50 thousand cells. Predicted affinity versus the length distribution for the peptides eluted from **(E)** 10 million, **(F)** 1 million, **(G)** 100 thousand and **(H)** 50 thousand cells. Black dots represent peptides with high predicted I-A^b^ binding affinity (IC_50_ <1µM), gray dots represent peptides low predicted I-A^b^ binding affinity (IC_50_ ≥1µM). Pie charts indicate the fraction of eluted peptides in each sample with predicted high and low binding affinities.

To systematically evaluate the effect of low-abundance sample amounts on the proportion of non-specific peptides detected in immunopeptidome workflows, we switched to the well-characterized human B-lymphoblastoid cell line LG2. These cells provide several advantages for validating methods to distinguish true MHC binders from non-specific peptides. LG2 cells have been used previously in many MHC-I and MHC-II immunopeptidome studies (29, 37–39), and large libraries of validated peptides are available. For example, in a recent study of the effect of HLA-DO on the MHC-II immunopeptidome we characterized >6000 DR1-bound peptides from this cell line (37). As in previous studies, in that work we used a large sample size of 100 million cells per preparation. LG2 cells are homozygous across the entire MHC, removing ambiguities often associated with HLA assignment common to studies of natural abundance MHC proteins in human cells. Finally, the peptide binding motif for HLA-DR1, the protein investigated here, is highly accurate in predicting experimental binding affinities (40), and can be used as an additional criterion to distinguish true binders from false positives. Here, we wanted to examine the effect of reduced sample sizes on non-specific peptide identification, and so we characterized the DR1 immunopeptidomes from LG2 samples of 10 million, 1 million, 100 thousand and 50 thousand cells (**Figure 3**). The length distributions of peptides eluted from 10 million, 1 million cells were centered around ~16 aa, with no peak around ~10 aa (**Figures 3A, B**). For the 100 thousand cells sample the median length was just slightly reduced to 15 aa, with a minor peak in the length distribution at 10 aa, indicating a small but appreciable level of non-specific peptides were present in these samples (**Figure 3C**). By contrast, the length distribution for the peptides eluted from 50 thousand cells showed very few peptides ~16 aa and a large number of shorter peptides, with an overall median length of ~ 11 aa, indicating that this sample was dominated by non-specific peptides (**Figure 3D**). Peptide binding preferences for DR1 are very well characterized (41) and we could confidently assign predicted peptide binding affinities to all of the eluted peptides using the NetMHCIIpan3.2 algorithm (with prediction performance of 0.83 as assessed by area under the receiver-operator curve analysis (42)). For the 10 million cell sample, >90% of the eluted peptides are predicted to bind with submicromolar affinity (**Figure 3E**, black symbols). As the number of cells analyzed is lowered, the fraction of peptides with predicted affinity <1000 nM decreases (**Figures 3F–H**). The peptides with low predicted affinity (**Figures 3E–H**, gray symbols) were dominated by short peptides with length ~9-11, similar to the non-specific peptides in the mouse samples, whereas the peptides predicted to bind more tightly with submicromolar predicted affinity had a length distribution centered around 16 aa (**Figures 3E–H**, black symbols), as expected for true DR1 binders (37). Thus, the amount of MHCII present in a sample can determine the fraction of non-specific peptides versus true MHC II binders in the pool of eluted peptides. As such, we used these datasets to investigate ways to parse out the specific binders present in low-abundance samples.

### Identifying True MHC-II Binders in Low Abundance Samples by Clustering Overlapping Peptides Into Core Epitopes

**Figure 4:**
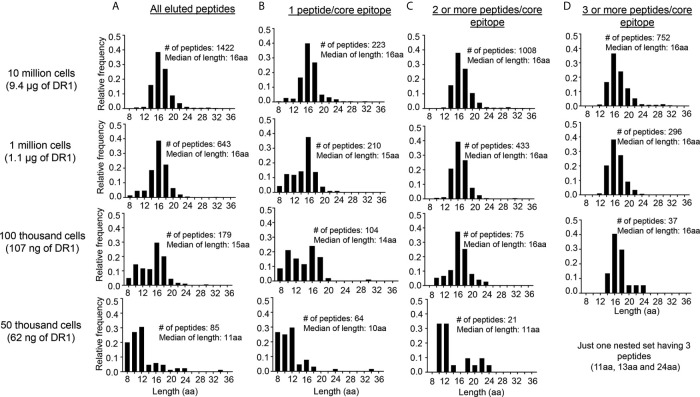
Length distribution of peptides eluted from LG2 cells with different numbers of sequences per core epitope. **(A)** All of the peptides eluted from 10 million, 1 million, 100 thousand and 50 thousand cells. NetMHCIIpan 3.2 was used for predicting the 9-residue binding frame from all peptides eluted from different numbers of LG2 cells. All peptides having identical predicted binding frames were considered as a single core epitope. **(B)** Length distribution for peptides having 1 peptide/core epitope from different numbers of LG2 cells. **(C)** Length distribution for peptides having 2 or more peptides/core epitope and **(D)** Length distribution for peptides having 3 or more peptides/core epitope. The number of peptides and median of length distribution for each set has been indicated.

Naturally-processed MHCII-bound peptides typically are found as nested sets with varying lengths centered around a core epitope, as a consequence of the MHC-II epitope generation pathway in which MHC-bound precursors are trimmed by endosomal endoproteases, leaving peptides with frayed ends that overhang the ~13aa-long MHC-II peptide binding site (43). We investigated whether this characteristic would be useful to help distinguish MHCII-bound from non-specific peptides present in the LG2 eluates, since non-specific peptides would not be expected to be present as nested sets surrounding a core epitope. We grouped eluted peptides with different lengths but having the same binding core (as predicted by NetMHCIIpan) into core epitopes. The fraction of peptides with only one peptide per core epitope increased from <16% for the 10 million cell sample (223 of 1422 total) to >75% for the 50,000 cell sample (64 of 85 total) (**Figures 4A, B**). The length distribution of peptides having only one peptide per core epitope included many shorter peptides, particularly in the 100,000 and 50,000 cell samples (**Figure 4B**). By contrast, the length distribution of the peptides having 2 or more peptides per core epitope was centered around ~16 aa for 10 million, 1 million and 100,000 cells, although the 50,000 cells sample still had many short peptides (**Figure 4C**). For peptides having 3 or more peptides per core epitope, essentially all the peptides had the expected length distribution, although for the 50,000 cells sample these peptides comprised only 1 nested set with three peptides (**Figure 4D**).

### Thymic I-A^b^ Epitopes Have Broader Length Distribution as Compared to Splenic B Cells and DC

**Figure 5:**
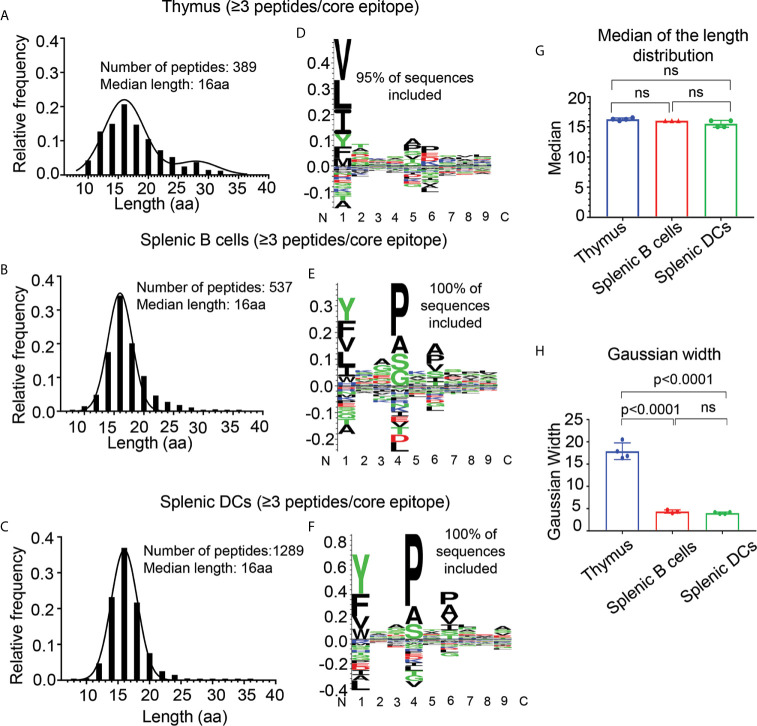
Length distribution **(A–C)** and motif analysis **(D–F)** for peptides present in nested sets of at least three peptides per core epitope, eluted from thymus, splenic B cells, and splenic DCs. **(A, D)** Eluted peptides from thymus; **(B, E)** from splenic B cells; and **(C, F)** from splenic dendritic cells (DCs). **(G)** Median of the length distribution for thymus, splenic B cells and DCs is ~16 aa for each biological replicate. **(H)** Gaussian width analysis for each the distribution of peptide lengths in each biological replicate, showing that the thymic peptides having broader length distribution as compared to splenic B cells and DCs. Motifs determined as in **Figure 2**. Mean ± SD are plotted, and p-values are calculated using unpaired t-test. The non-significant differences are denoted as ns.

Having validated the strategy of clustering peptides into core epitopes using the human LG2 cell line, we switch back to analysis of the peptidomes eluted from thymus, splenic B cells and splenic DCs (**Figure 5** and **Supplementary Table S3**). Similarly to the LG2 studies described above, the fraction of peptides present with only one peptide per core epitope decreased with increasing amounts of I-A^b^ in the sample, whereas the reverse trend was observed for peptides present at two or more per core (**Supplementary Figure S5**). Considering only the peptides present as 3 or more peptides per core epitope, the number of shorter peptides was considerably reduced in each sample as compared to the total peptidomes (**Figures 5A–C**, cf. **Figures 2A–C**), and all of the samples had length distributions centered around 16 aa (**Figure 5G**). Again, considering only peptides present at 3 or more peptides per core epitope, sequence motifs for the splenic B cell and DC peptidomes showed the strong concentration of hydrophobic amino acid residues at the P1 and small residues at the P4 and P6 positions (**Figures 5E, F**). The thymic peptidome sequence motif had similar preferences at P1, but the P4 and P6 preferences were more muted (**Figure 5D**). We used the 3+ peptides per core epitope datasets to begin to investigate differences in antigen processing and presentation pathways between thymus and spleen. Despite the similar median lengths, the shape of the peptide length distribution was much broader for the thymic peptidome as compared to splenic B cell and DC peptidomes (**Figures 5G, H**). There appears to be a significantly longer component with median length 28 residues present specifically in the thymus, but even excluding that component the width of the main peak centered around 16 aa is broader for the thymus than for the splenic samples.

### Differences Between I-A^b^-Presented Peptidome of Thymus, Splenic B Cells, and Splenic DCs

**Figure 6:**
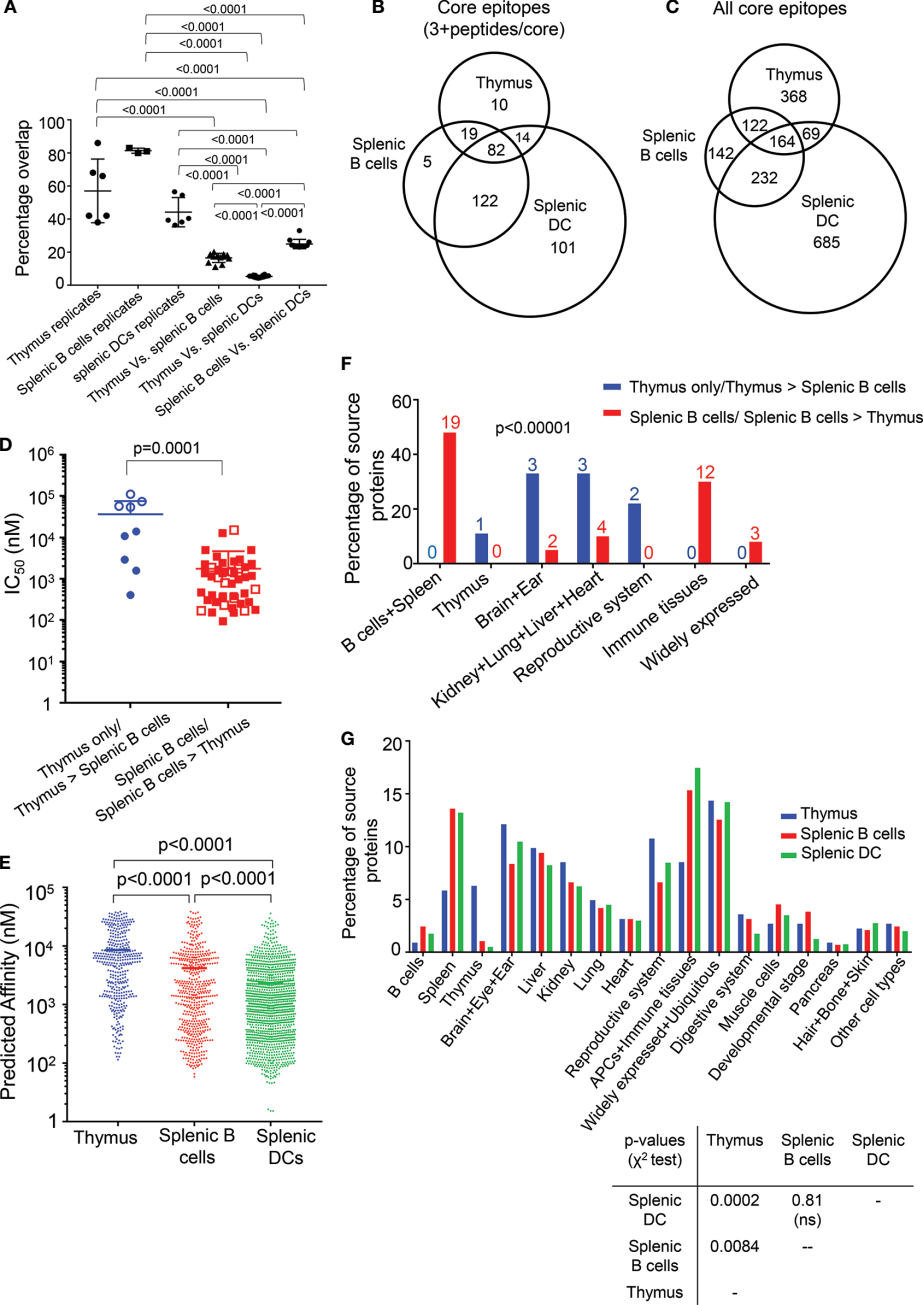
Overlap, affinity, and tissue distribution analysis for peptides eluted from thymus, splenic B cells, and splenic DC. **(A)** The percentage overlap of core epitopes between replicates of thymus, splenic B cells and DCs is shown, along with the percentage overlap between the different cell types. The p values shown represent differences in the percentage overlap for within-sample and between-sample values, and for differences between thymus and splenic B and DCs. The mean ± SD are plotted, and the p values are calculated using unpaired t test. **(B)** Venn diagram showing the overlap between the core epitopes of thymus, splenic B cells and DCs, for epitopes represented by at least three nested peptides. **(C)** As **(B)**, but for all core epitopes. **(D)** The experimental I-A^b^ binding affinity was determined for the most abundant peptide from each differentially presented core epitope having at least three peptides per core. Filled blue circles show epitopes present in at least three of the four biological replicates of thymus and absent from all replicates of splenic B cells, open blue circles show epitopes present in at least three of the four biological replicates of thymus and present with at least 2-fold higher amounts in thymus as compared to splenic B cells, filled red squares show epitopes present in all three biological replicates of splenic B cells and absent from all replicates of thymus, and open red squares show epitopes present in all three biological replicates of splenic B cells and present with at least 2-fold higher amounts in in splenic B cells as compared to thymus. The peptides present only in the splenic B cells or in higher abundance in splenic B cells have higher affinity as compared to the peptides present only in thymus or in higher abundance in thymus. Binding affinities are reported as IC_50_ values. The p values were calculated using Mann-Whitney test. **(E)** Predicted affinity for peptides present in nested sets of at least three peptides per core epitope, eluted from thymus, splenic B cells and splenic DCs. The predicted affinity for the peptides eluted from the DCs are of higher than the splenic B cells and the peptides eluted from the splenic B cells have higher predicted affinity as compared to the thymic peptides. The p values were calculated using Mann-Whitney test. **(F)** The tissue sources for the proteins of the peptides from panel **(D)** differentially present in thymus (blue) or splenic B cells (red) were identified using the Uniprot database. The immunopeptidome from the brain specific proteins, reproductive system proteins, heart, ear, lung and kidney are found to be enriched in thymus. The p values were calculated using Fisher’s exact test. **(G)** Tissue sources for all the source proteins eluted from the thymus, splenic B cells and splenic DCs samples and that have ≥ 2 peptides/source protein were analyzed using the Uniprot database. The p-values between each set are indicated and were calculated using chi-square test.

To explore differences between the immunopeptidomes of thymus, splenic B cells and DCs, we performed an overlap analysis to identify core epitopes shared or uniquely presented in the various cell types. We use the same parsed datasets as in **Figure 5**, only considering peptides present as 3 or more peptides per core epitope, but for the overlap analysis we analyzed core epitopes rather than the individual peptides. The pairwise between-sample overlap was 25% for comparisons of splenic B and DC samples, but only 17% and 5% for comparison of the thymic samples with either splenic B or DC respectively (**Figure 6A**). The lowest overlap was found for the thymus and splenic DC samples, despite the larger number of peptides present in DC samples (**Table 1**). By contrast, the within-sample core epitope overlap between biological replicates was much larger, varying from an average of ~44% for the DC samples to ~81% for the splenic B cells (**Figure 6A**). Thus, the smaller overlaps seen between thymus and splenic B cells, or between thymus and splenic DC, likely reflect substantial differences in these immmunopeptidomes. These differences between immunopeptidomes from the various cell types also are apparent also in Venn diagrams showing the distribution of core epitopes with 3+ peptides among the different samples (**Figure 6B**, **Supplementary Table S4**), and the distribution of all core epitopes (**Figure 6C** and **Supplementary Table S4**).

To see if the somewhat less distinct sequence motif observed for the thymic peptidome as compared to the splenic B cell and DC peptidomes was accompanied by reduced MHC-peptide binding affinity, we evaluated I-A^b^ binding activity. For this purpose, we compared thymic and splenic B cell peptidomes, because for these datasets the thymus and spleen preparation came from the same mice and were processed and analyzed in parallel. To minimize the effects of sample-to-sample variation we considered only core epitopes present in most of the biological replicates from thymus (at least 3 of the four samples) or all of the biological replicates of splenic B cells (three samples). As in the previous analysis, we considered only core epitopes present in three or more peptides, to minimize any possible effect of non-specific peptides. For each core epitope we synthesized the most abundant peptide sequence carrying that core, and measured the relative binding affinity using purified recombinant I-A^b^ and an *in vitro* fluorescent peptide competition binding assay (34), reporting the results as IC_50_ values (**Figure 6D**). We compared core epitopes presented preferentially in thymus (**Figure 6D**, blue symbols) with those presented preferentially in splenic B cells (red symbols), including those found exclusively (filled symbols) or in at least 2-fold greater abundance (open symbols) in each sample type. The thymic epitopes were characterized by lower binding affinity (average IC_50_ = 37 µM) as compared to the splenic B cell epitopes (1.7 µM), and spanned a wider range of affinities despite being represented by fewer epitopes (**Figure 6D** and **Supplementary Table S5**). Since examining just the outlier core epitopes uniquely present only in thymus or splenic B might exaggerate differences between the samples, and to examine the generality of these results in the full immunopeptidome datasets, we used a computational approach, predicting IC_50_ values for all the peptides eluted from thymus, splenic B cells, or splenic DC, using the NetMHCIIpan 3.2 algorithm. The average predicted binding affinities of all the peptides eluted from the three different cell types were significantly different, with the DC peptides having the highest average predicted affinity (**Figure 6E**). As for the experimental determination in **Figure 6D**, the full set of thymic peptides had predicted affinity lower than the splenic B cells peptides.

Finally, we compared the tissue distribution of source proteins for core epitopes eluted from the various cell types. First, we compared the closely matched thymic and B cell samples, identifying the typical physiological location or tissue expression of the source proteins for each of the differentially presented core epitopes as in **Figure 6D**. Both B cell and thymic epitopes derived from a wide range of tissue sources. The representation of thymic peptides was significantly greater than B cell peptides for source proteins from several tissues, including brain, ear, and reproductive system, but less in other tissues (**Figure 6F**). This pattern was apparent, although to a lesser degree, for the full set of source proteins (represented by at least two eluted peptides) from all tissue sources (**Figure 6G**).

## Discussion

To explore possible differences in MHCII antigen processing and presentation pathways between thymus and spleen, we characterized the I-A^b^-presented peptidomes from these tissues in C57BL/6 mice. We evaluated several strategies to reduce the contribution of non-specific peptides that appeared to dominate the thymus sample, using a well-characterized human B-lymphoblastoid cell line as a test system. Limiting the analysis only to peptides present in nested sets surrounding a core epitope essentially eliminated the contribution of non-specific peptides in the peptidomes. Even with the consequent reduction in the number of peptides identified, we nonetheless identified >400 peptides from mouse thymus present in nested sets of at least three peptides. This represents a substantial improvement over previous description of mouse and human thymic immunopeptidomes (8–10). We observed substantial differences in peptide length distribution and binding affinity for the thymic as compared to splenic peptidomes, suggestive of differences in the stringency of antigen processing and editing in these tissues.

In initial studies we observed many apparently non-specific peptides present in the thymic samples. We found that removal of peptides identified in control elutions from isotype and bead-only experiments was useful in reducing this background, however many remained, as indicated by peptide length distributions and sequence motifs inconsistent with known I-A^b^ features. The same behavior was observed for test human cell line samples when assayed at very low abundance, where we could unambiguously identify non-specifically-bound peptides. Why didn’t the isotype-control approach to background subtraction, routinely applied in flow cytometry and cell biology applications, work in this case? We suspect that a contributing factor is the stochastic nature of data-dependent acquisition pipelines, wherein selection of peptide ions for fragmentation and sequence determination is directed at identification of a greater number of species rather than greater reproducibility between samples, with the result that low abundance species are difficult to reproducibly detect across samples. Using different strategies for identification of non-specifically bound peptides, including length cutoffs, predicted MHC binding affinity, and absence from nested sets, we identified non-specific peptides with characteristics similar to those eluted from the control affinity beads, although the particular sequences identified were different. A previous approach employing the PLAtEAU algorithm (44) used sequence overlaps rather than shared MHC-binding cores to identified peptides in nested sets; that approach likely would also reduce any contribution from non-specifically bound peptides as we observe here. For our purposes in this study, consideration of peptides present only in nested sets provided a convenient approach to reduction of the contribution of these non-specifically bound peptides. However, this approach is likely to remove some specifically bound peptides, particularly those present at low abundances, and may not be appropriate for other immunopeptidome study designs or goals. We note that this approach would not be appropriate for most class I MHC proteins, which typically do not bind nested sets of peptides because of constraints in the MHC-I peptide binding site with binding sites for both peptide N- and C- termini. Strategies employed in other studies should help to mitigate this issue, including imposition of a length-cutoff, predicted affinity cutoff, or consideration of peptides present only in most or all biological or technical replicates (44), with the choice of strategy guided by the goals of the study and the relative enrichment of specific versus non-specific species.

The problem of identification of non-specific peptides present in an eluted peptide sample is distinct from that addressed by measures to control the false discovery rate, which seek to reduce the frequency of incorrect identification of peptide sequences in the database matching algorithm. Here, the issue is one of biochemical specificity; the peptides appear to be identified correctly and are present in the samples but are not specifically bound by the MHC protein of interest. Possible sources for such peptides include co-purifying peptide-binding proteins such as heat-shock proteins, scavenger receptors, or potentially even MHC-I proteins, as well as exogenously or endogenously processed peptides that bind directly to the immunoaffinity resin. All protein purification procedures have a finite purification ratio and entail some degree of sample loss. In attempting to increase recovery of the desired MHC-bound peptides while minimizing undesired non-specific peptides, a balance must be struck between sensitivity and specificity. Additional optimization of our isolation strategy might improve its performance, particularly for low-abundance samples. However, non-specific peptides likely will be present to some extent in all immunopeptidome samples, particularly those for which the MHC-peptide content is low, but how to address these non-specifically isolated co-purifying peptides seems not to have been addressed specifically in the literature. Strategies employed in other studies should help to mitigate this issue, including imposition of a length-cutoff, predicted affinity cutoff, or consideration of peptides present only in most or all biological or technical replicates, with the choice of strategy guided by the goals of the study and the relative enrichment of specific versus non-specific species.

Despite considerable overlap between the thymic and splenic peptidomes, almost 2/3 of the confidently identified MHC-II core epitopes from thymus, i.e. those present in at least three nested peptides, were not found in the splenic B cell or DC elutes. Among the thymus-specific peptides were many deriving from source proteins expressed from tissue-specific genes, including those typically expressed in brain, skin, eyes, etc. In previous studies of human thymus, a few such peptides presented by MHC-I or MHC-II proteins were identified and associated with Aire-dependent transcription in medullary thymic epithelial cells (mTEC) (10, 45). Whether thymus-specific peptides that we identified are Aire-dependent transcripts expressed in mTEC or represent true tissue-specific proteins brought into thymus by migratory DC, B cells, or other antigen presenting cells requires further work, possibly including fractionating the various cells types present in thymus before elution, as previously reported for murine thymus CD11c+ DC in a study of MHC-I peptidomes (9). In addition to differences in source protein distribution, we observed substantial differences in length distribution and I-A^b^ binding affinity for the I-A^b^-binding peptides eluted from thymus compared to splenic DC and B cells. The thymic immunopeptidome had a broader distribution of lengths than the splenic peptidomes, possibly indicative of less aggressive trimming by cellular proteases, and a broader distribution of I-A^b^ affinity. The affinity differences might indicate that thymic peptides have undergone less efficient editing by DM, a non-classical MHC protein known to select for tightly-bound MHCII-peptide complexes (46, 47). Thymic epithelial cells express abundant DO, another non-classical MHC protein that inhibits DM (48) and its expression increases presentation of low affinity peptides (37). The less aggressive processing and less efficient editing characteristics of antigen presentation in the thymus might help to preserve many delicate, rare, or low-affinity epitopes for presentation to developing thymocytes during selection in the thymus.

One limitation of our study is that absolute quantitation approaches, such as internal standard doping with known amounts of stable-isotope-labeled eluted-peptide variants (37) were not performed, so that comparisons of the abundances of different peptides are not available. Another limitation is our reliance on datasets obtained by data-dependent acquisition workflows, which are optimized for identifying as many peptides as possible. If larger spectral libraries were available, a data-independent (DIA) approach, as recently applied to low-abundance human blood MHC-II peptidomes (49), could allow for better control of sample-to-sample differences. As in other studies of limited-abundance biological samples (10, 49, 50), our conclusions are constrained by the small size of the available immunopeptidomes. Finally, tagging approaches that allow for more efficient MHC purification might reduce the impact of non-specific peptides (51).

In summary, our initial efforts to characterize the peptides presented by I-A^b^ in the thymus of C57BL/6 mice using conventional immunopeptidome sample processing and analysis pipelines were complicated by the presence of a substantial fraction of non-specifically bound, co-purifying peptides, apparently the result of the low levels of I-A^b^ present in this tissue. We used a well-characterized human cell line, for which HLA-DR-binding peptides could easily be identified, to test several strategies to reduce the contribution of background peptides. By considering only peptides present in nested sets surrounding a core epitope, we distinguished binders from background peptides, in low abundance human and mouse thymus samples. Comparison of the mouse thymus immunopeptidome with those of mouse splenic B cells or DC suggested that antigen presentation pathways in the thymus are characterized by less aggressive processing and less stringent editing than those in the spleen.

## Data Availability Statement

The datasets presented in this study can be found in MassIVE data repository (http://massive.ucsd.edu) developed by Center for Computational Mass Spectrometry (University of California, San Diego) with the project accession MSV000087031.

## Ethics Statement

The animal study was reviewed and approved by University of Massachusetts Medical School Institutional Animal Care and Use Committee.

## Author Contributions

PN and LJS designed the research. PN isolated peptidomes, performed binding studies, and analyzed mass spectrometry data. MJ isolated thymus, splenic B cells, and dendritic cells. CC isolated dendritic cells. LL assisted in recombinant protein purification. PN, LS, and LJS analyzed the data and wrote the paper. All authors contributed to the article and approved the submitted version.

## Funding

This work was supported by NIH grant R01AI137198 (LS and LJS).

## Conflict of Interest

The authors declare that the research was conducted in the absence of any commercial or financial relationships that could be construed as a potential conflict of interest.
