# Supplementary material for: Distinguishing Signal From Noise in Immunopeptidome Studies of Limiting-Abundance Biological Samples: Peptides Presented by I-Ab in C57BL/6 Mouse Thymus
Source: Front Immunol. 2021 Apr 29;12:658601. doi: 10.3389/fimmu.2021.658601 (PMC8116589; doi:10.3389/fimmu.2021.658601)
Supplement: Supplementary file 1 [file DataSheet_1.pdf]

List of Supplementary Materials

Supplementary Figure S1. Length distribution and the motif analysis for control peptides eluted from beads-only and isotype-control antibody columns.

Supplementary Figure S2. Motif analysis for thymic peptides of each length.

Supplementary Figure S3. Length distribution for peptides eluted from total membrane fraction of thymus samples.

Supplementary Figure S4. Length distribution for peptides eluted from thymus that were observed in all three biological replicate samples, with each sample having at least two of three technical replicates.

Supplementary Figure S5. Fraction of peptides in nested sets with one, two, or three peptides per core epitope as a function sample I-A^b amount.

Supplementary Table S1. MHC-peptide complex stepwise yields after the Dounce homogenization and solubilization and preclearing steps including unconjugated beads and isotype control antibody beads.

Supplementary Table S2. Details of the eluted peptides from all biological replicates of thymus, splenic B cells, and splenic DCs. Columns show source protein information, peptide sequence, predicted core epitope (as identified by NetMHCIIpan3.2 for peptides with at least 9 residues), and ion intensity values for each technical and biological replicate. Separate sheets show information for thymus, splenic B cells, and splenic DC.

Supplementary Table S3. Details of the eluted peptides having ≥3 peptides/core.

Supplementary Table S4. Distribution of core epitopes between thymus, splenic B, and splenic DC samples. Sheet 1 shows the core epitopes presented by three or more peptides per nested set for the thymus, splenic B cells and splenic DCs samples. Sheet 2 shows the same information for all core epitopes.

Supplementary Table S5. Experimental I-A^b binding affinity IC_{50} values for the differentially expressed peptides.

Supplementary Table S6. List of the non-specific peptides eluted from the beads-only and isotype-control antibody columns from the thymus preparations.

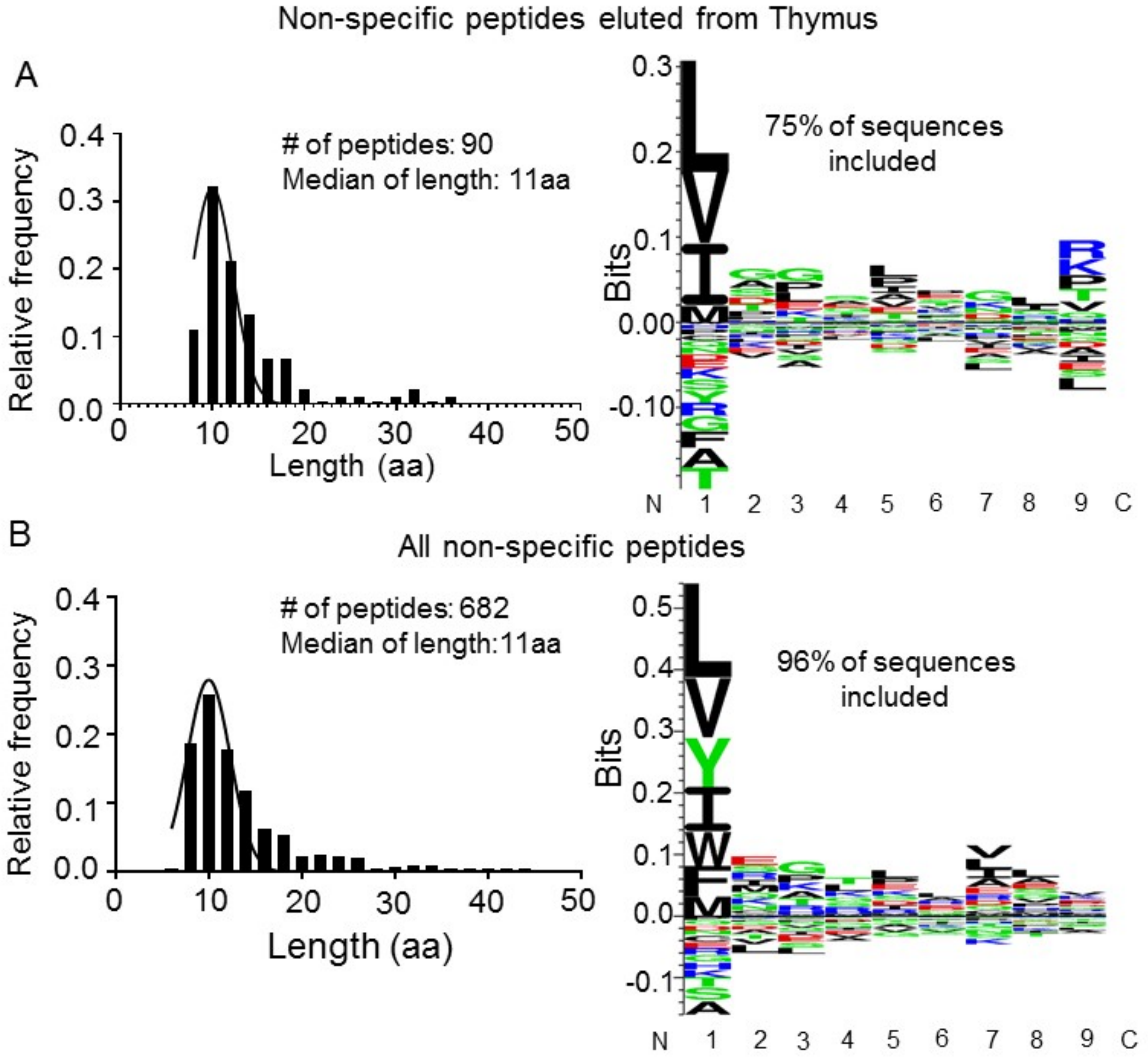

Supplementary Figure S1. Length distribution and the motif analysis for control peptides eluted from beads-only and isotype-control antibody columns. A) Control peptides eluted from thymus samples. B) Control peptides eluted from all the cell types.

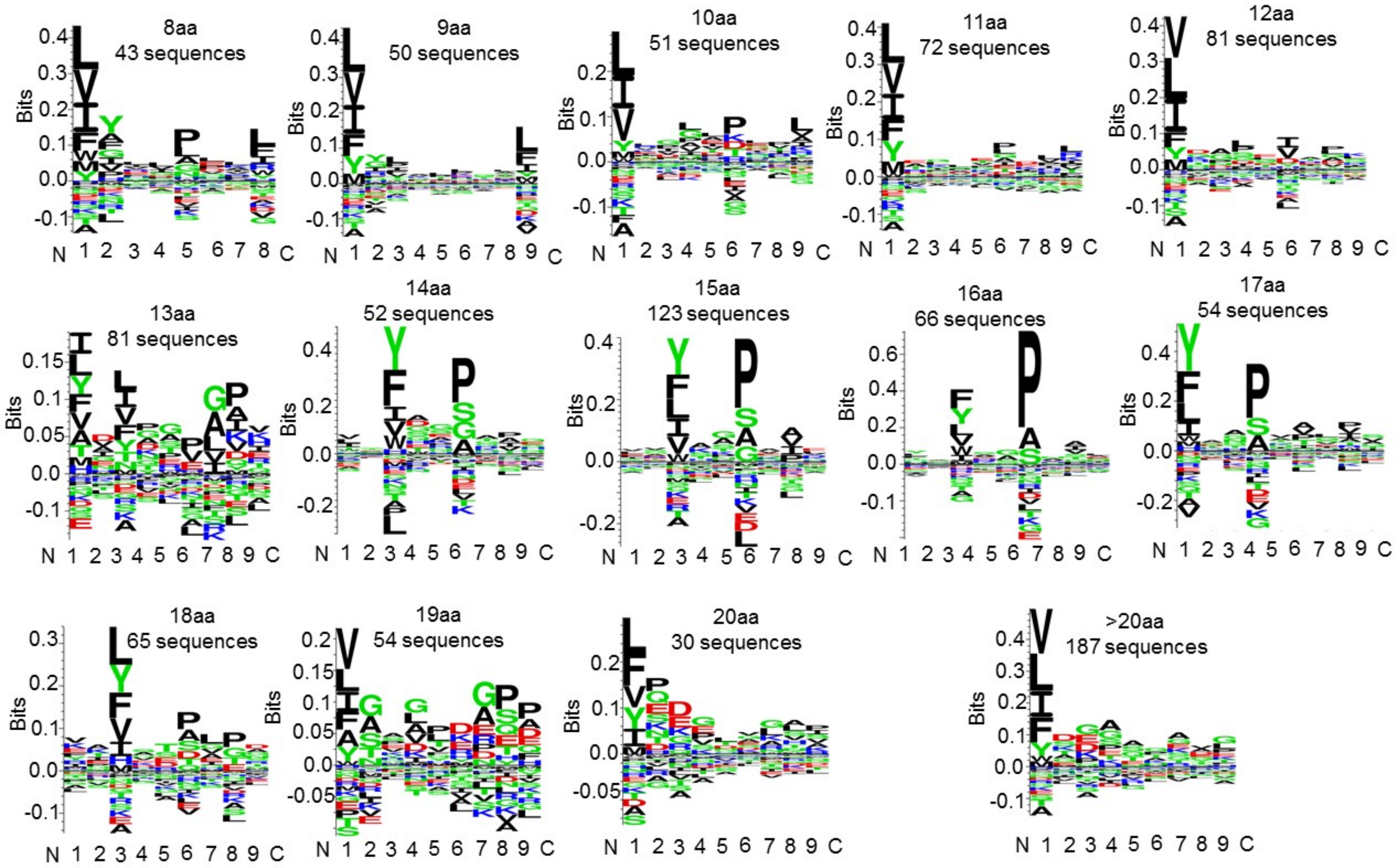

Supplementary Figure S2. Motif analysis for thymic peptides of each length.

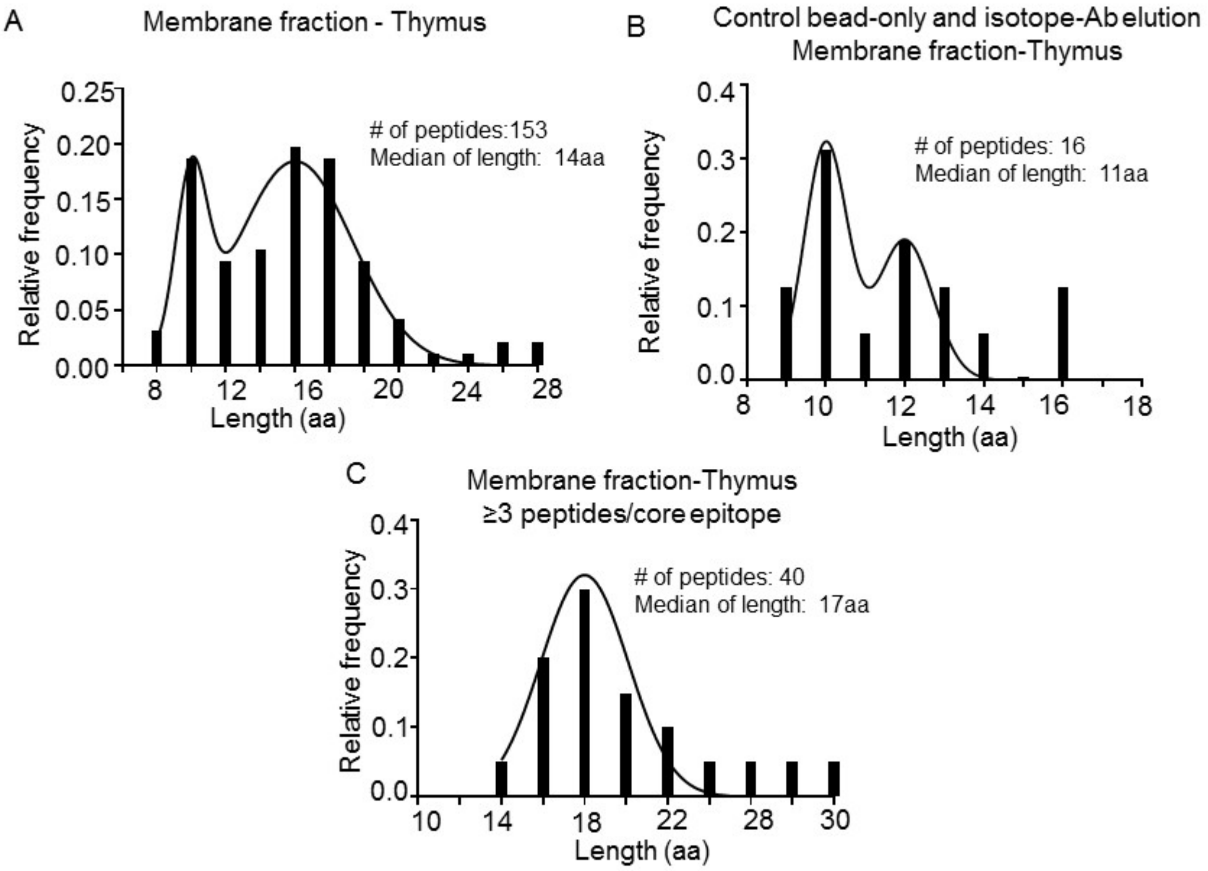

Supplementary Figure S3. Length distribution for peptides eluted from total membrane fraction of thymus samples. A) Peptides eluted from I-A^b-specific antibody column. B) Peptides eluted from control beads-only and isotype-control antibody columns. C) Peptide eluted from I-A^b-specific antibody column as in panel A, but only for those present in nested sets of at least 3 peptides per core epitope.

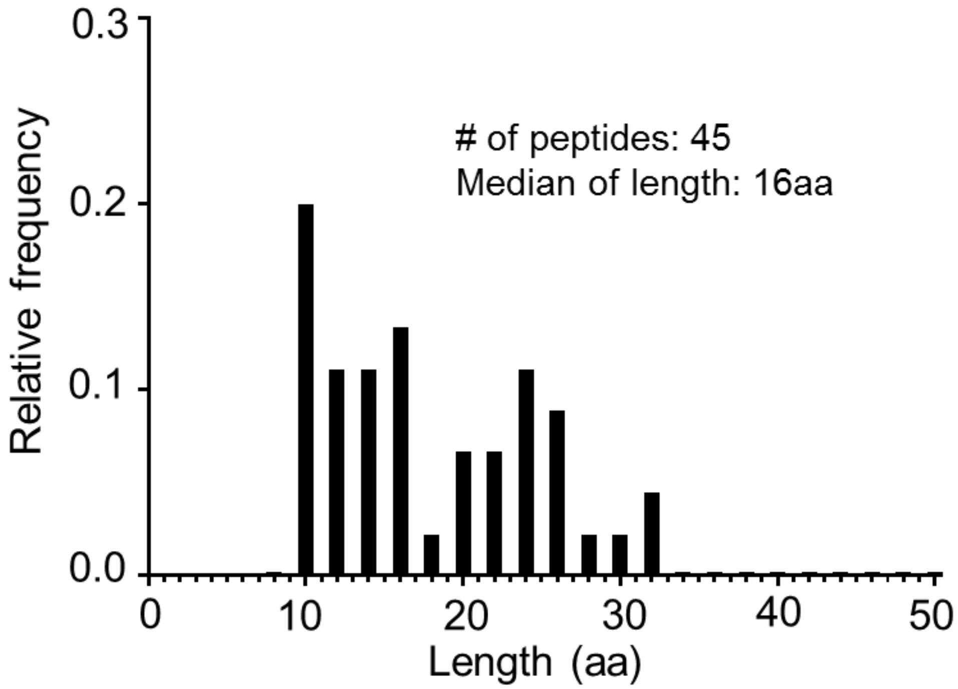

Supplementary Figure S4. Length distribution for peptides eluted from thymus that were observed in all three biological replicate samples, with each sample having at least two of three technical replicates.

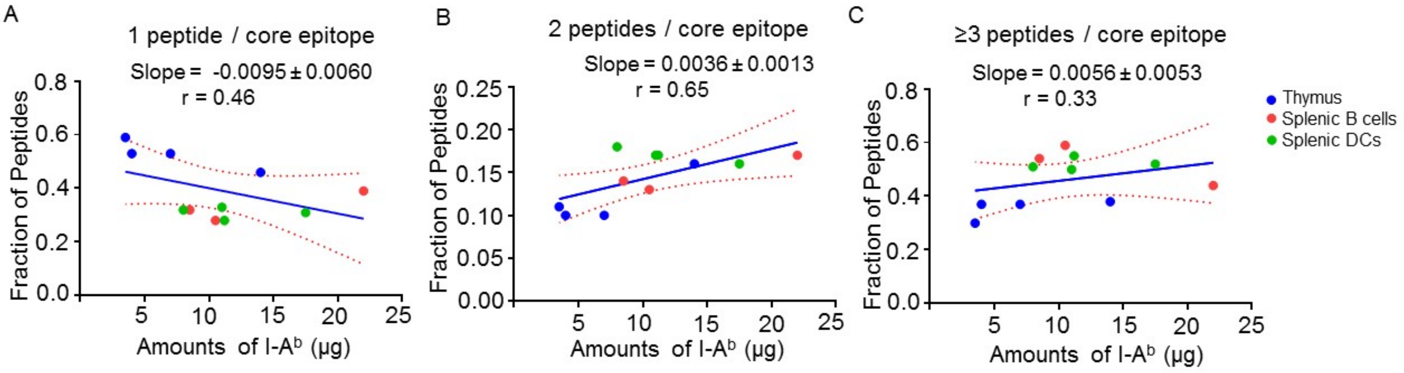

Supplementary Figure S5. Fraction of peptides in nested sets with one, two, or three peptides per core epitope as a function sample I-A^b amount. Fraction of total number of peptides represented by A) 1 peptide/core epitope, B) 2 peptides/core, and C) ≥3 peptides / core are plotted against the amount of I-A^b in each sample of thymus (blue symbols), splenic B cells (red), and splenic DCs (green). Pearson correlation r values are indicated along with the slopes calculated using linear regression. Standard error of slope values is shown.
